# Role of anoctamin-1 and bestrophin-1 in spinal nerve ligation-induced neuropathic pain in rats

**DOI:** 10.1186/s12990-015-0042-1

**Published:** 2015-07-01

**Authors:** Jorge Baruch Pineda-Farias, Paulino Barragán-Iglesias, Emanuel Loeza-Alcocer, Jorge E Torres-López, Héctor Isaac Rocha-González, Francisca Pérez-Severiano, Rodolfo Delgado-Lezama, Vinicio Granados-Soto

**Affiliations:** Neurobiology of Pain Laboratory, Departamento de Farmacobiología, Centro de Investigación y de Estudios Avanzados (Cinvestav), Sede Sur, Calzada de los Tenorios 235, Colonia Granjas Coapa, 14330 México, D.F., México; Departamento de Fisiología, Biofísica y Neurociencias, Centro de Investigación y de Estudios Avanzados (Cinvestav), Zacatenco, México, D.F., México; Laboratorio de Mecanismos de Dolor, División Académica de Ciencias de la Salud, Universidad Juárez Autónoma de Tabasco, Villahermosa, Tabasco México; Hospital Regional de Alta Especialidad “Dr. Juan Graham Casasús”, Villahermosa, Tabasco México; Sección de Estudios de Posgrado e Investigación, Escuela Superior de Medicina, Instituto Politécnico Nacional, México, D.F., México; Departamento de Neuroquímica, Instituto Nacional de Neurología y Neurocirugía “Manuel Velasco Suárez”, México, D.F., México

**Keywords:** Allodynia, Anoctamin-1, Bestrophin-1, Calcium-activated chloride channels, Neuropathic pain, Spinal nerve ligation

## Abstract

**Background:**

Calcium-activated chloride channels (CaCCs) activation induces membrane depolarization by increasing chloride efflux in primary sensory neurons that can facilitate action potential generation. Previous studies suggest that CaCCs family members bestrophin-1 and anoctamin-1 are involved in inflammatory pain. However, their role in neuropathic pain is unclear. In this investigation we assessed the involvement of these CaCCs family members in rats subjected to the L5/L6 spinal nerve ligation. In addition, anoctamin-1 and bestrophin-1 mRNA and protein expression in dorsal root ganglion (DRG) and spinal cord was also determined in the presence and absence of selective inhibitors.

**Results:**

L5/L6 spinal nerve ligation induced mechanical tactile allodynia. Intrathecal administration of non-selective CaCCs inhibitors (NPPB, 9-AC and NFA) dose-dependently reduced tactile allodynia. Intrathecal administration of selective CaCCs inhibitors (T16A_inh_-A01 and CaCC_inh_-A01) also dose-dependently diminished tactile allodynia and thermal hyperalgesia. Anoctamin-1 and bestrophin-1 mRNA and protein were expressed in the dorsal spinal cord and DRG of naïve, sham and neuropathic rats. L5/L6 spinal nerve ligation rose mRNA and protein expression of anoctamin-1, but not bestrophin-1, in the dorsal spinal cord and DRG from day 1 to day 14 after nerve ligation. In addition, repeated administration of CaCCs inhibitors (T16A_inh_-A01, CaCC_inh_-A01 or NFA) or anti-anoctamin-1 antibody prevented spinal nerve ligation-induced rises in anoctamin-1 mRNA and protein expression. Following spinal nerve ligation, the compound action potential generation of putative C fibers increased while selective CaCCs inhibitors (T16A_inh_-A01 and CaCC_inh_-A01) attenuated such increase.

**Conclusions:**

There is functional anoctamin-1 and bestrophin-1 expression in rats at sites related to nociceptive processing. Blockade of these CaCCs suppresses compound action potential generation in putative C fibers and lessens established tactile allodynia. As CaCCs activity contributes to neuropathic pain maintenance, selective inhibition of their activity may function as a tool to generate analgesia in nerve injury pain states.

**Electronic supplementary material:**

The online version of this article (doi:10.1186/s12990-015-0042-1) contains supplementary material, which is available to authorized users.

## Background

Neuropathic pain occurs as a direct consequence of either a lesion or disease affecting the somatosensory system [1]. Under such conditions, pain can be spontaneously elicited by either normally innocuous stimuli (allodynia) or by noxious stimuli (hyperalgesia) [2–4]. These conditions stem from enhanced sensory excitability. They are attributable to peripheral or central sensitization caused by either increased synaptic excitation, decreased synaptic inhibition (disinhibition), increased neuronal responsiveness, or any combination thereof [5]. An important component in nerve injury-induced hypersensitivity is pain-related information amplification resulting from expression and modulation of specific ion channels in the periphery and spinal dorsal horn [6]. However, the molecular mechanisms underlying this plasticity are not fully understood.

Calcium-activated chloride channels (CaCCs) include anoctamins, also known as TMEM16 (Ano-1 to Ano-10) [7–9] and bestrophins (Best-1 to Best-3) [10–12] families. Anoctamins and bestrophins are widely expressed in a host of tissues, which suggests their involvement in multiple physiological functions. Indeed, CaCCs participate in phototransduction, olfactory transduction, smooth muscle contraction, epithelial secretion and neuronal excitability [13–18]. Regarding the latter, anoctamins [19–21] and bestrophins [22–24] are present in sensory neurons and their activation, by an increase in intracellular Ca^2+^, elicits chloride efflux [13, 15, 25, 26]. Therefore, it has been suggested that CaCCs may promote depolarization of nociceptive terminals and they might be a key factor in the generation of action potentials [27]. In support of this idea, electrophysiological studies demonstrated that anoctamin-1 augments excitability and contributes to depolarization of dorsal root ganglia (DRG) neurons [19–21], while sciatic nerve axotomy enhances bestrophin-1 expression and calcium-activated chloride currents in DRG neurons [23, 28] suggesting the participation of CaCCs in neuropathic pain. Accordingly, it has been reported that anoctamin-1 ablation reduces nociceptive behavior after spared nerve injury [21]. However, so far there has not been any systematic evaluation about the role of CaCCs in spinal nerve injury-induced neuropathy. Accordingly, we report here on the role of CaCCs in neuropathic pain induced by L5/L6 spinal nerve ligation.

## Methods

### Animals

Adult female Wistar rats (140–160 g, 6–7 weeks) from our own breeding facilities were used in this study. Female rats were used based on the fact that previous studies from our laboratory have found no differences in tactile allodynia between female and male rats [29]. These animals were housed in a controlled environment, on a 12-h light/dark cycle, with free access to food and water. All experiments were conducted according to the National Institutes of Health Guide for Care and Use of Laboratory Animals (Publication No. 85-23, revised 1985), Guidelines on Ethical Standards for Investigation of Experimental Pain in Animals [30] and were approved by our local Ethics Committee (Protocol 0042-13, Cinvestav, Mexico City, Mexico). In addition, every effort was made to minimize pain and suffering, and the number of rats used was the least required to obtain significant statistical power.

### Induction of nerve injury and measurement of tactile allodynia

Neuropathic pain was induced by spinal nerve ligation [31]. Briefly, rats were anesthetized with a mixture of ketamine (50 mg/kg, i.p.) and xylazine (10 mg/kg, i.p.). After surgical preparation and exposure of the dorsal vertebral column, the left L5 and L6 spinal nerves were exposed and tightly ligated with 6-0 silk suture distal to the DRG. For sham-operated rats, the nerves were exposed but not ligated. Rats exhibiting motor deficiency such as paw dragging were discarded from the study.

Tactile allodynia was determined as previously described [32]. Fourteen days after surgery, animals were placed in cages with a mesh grid floor and allowed to acclimate for a minimum of 30 min before performing the experiment. Von Frey filaments (Stoelting, Wood Dale, IL, USA) were used to determine the 50% paw withdrawal threshold using the up-down method of Dixon [33]. A series of filaments, starting with one that had a buckling weight of 2 g, were consecutively applied in consecutive sequence to the plantar surface of the right hind paw with a pressure causing the filament to buckle. Lifting of the paw indicated a positive response and prompted the use of the next weaker filament, whereas absence of paw withdrawal after 5 s indicated a negative response and prompted the use of the next heavier filament in a series. This paradigm continued until four more measurements were made after the initial change of the behavioral response or until five consecutive negative (assigned a score of 15 g) or four consecutive positive (assigned a score of 0.25 g) responses occurred. The resulting scores were used to compute the 50% withdrawal threshold by using the formula: 50% g threshold = 10^(Xf +κδ)^/10,000, where Xf = value (in log units) of the final von Frey filament used, κ = the value from table published by Dixon [33] for the pattern of positive and/or negative responses, and δ = the mean difference (in log units) between stimulus strengths. Allodynia was considered to be present when paw withdrawal thresholds were less than 4 g.

### Thermal hyperalgesia

The Hargreaves test measured the latency to radiant heat using a paw thermal stimulator [34]. Briefly, rats were placed individually in Plexiglass cubicles and allowed to acclimate for 20–30 min before the experiment. A radiant heat stimulus was applied to the base of the paw as it rested on a glass plate maintained at 30 ± 0.1°C at 0, 1, 2, 4, 6 and 8 h after drug administration. A timer was automatically actuated when the light source was turned on. The response latency was defined as the time required for abrupt withdrawal of the paw. In all cases, a cut-off of 20 s was employed to avoid tissue injury. Each test was repeated three times and the average paw withdrawal latency was calculated based on three measurements.

### Assessment of motor activity

Motor coordination was assessed in the Rotarod system [35]. Animals were acclimated 2 days before evaluation by walking them each day on an accelerating Rotarod apparatus (Panlab Harvard Apparatus, Barcelona, Spain). On the third day, animals were tested starting 1.5 h before and immediately after drug administration as well as 1.5 and 3 h later (time of peak drug effect). Evaluation was carried out with an acceleration from 4 to 40 rpm in 10 min. Motor coordination was considered as the time latency to fall off the Rotarod apparatus. It was determined from the mean time in three trials for each rat at each time.

### Spinal catheterization

Seven days after spinal nerve ligation surgery, rats were again anesthetized with a ketamine (50 mg/kg, i.p.) and xylazine (10 mg/kg, i.p.) combination and placed in a stereotaxic head holder in order to expose the atlanto-occipital membrane [36]. After piercing the membrane, a PE-10 catheter (7.5 cm) was passed intrathecally to the level of the thoracolumbar junction and the wound was sutured. Rats were allowed to recover from surgery for 7 days in individualized cages before use. Animals showing any signs of motor impairment were eliminated from the study and euthanized with a CO_2_ chamber.

### Semi-quantitative reverse transcription-polymerase chain reaction

Total RNA from ipsilateral (injured side) dorsal spinal cord segments L1–S1 as well as ipsilateral DRG (L4–L6) were isolated by the guanidinium-thiocyanate method with TRIzol-reagent according to the manufacturer’s instructions (Invitrogen Corporation, Carlsbad, CA, USA). The concentration and purity of the RNA were determined by measuring the absorbance at 260 and 280 nm in a NanoDrop ND-2000 spectrophotometer (Thermo Fisher Scientific, Wilmington, DE, USA). Equal amounts (5 μg) of RNA from samples were reversed-transcribed into first-strand complementary DNA (cDNA) using oligo dT and M-MLV-reverse transcriptase (Invitrogen Corporation, Carlsbad, CA, USA). The reaction was performed at 37°C for 60 min, followed by a heat denaturation step at 95°C for 5 min. Amplification of cDNA by polymerase chain reaction (PCR) was performed with specific primers previously reported [23] for bestrophin-1 (forward, 5′-TGGCAGAACAGCTCATCAAC-3′ and reverse, 5′-GCTGCCTCGTTCCAGTACAT-3′, 100 bp product) and anoctamin-1 (forward, 5′-TTCGTCAATCACACGCTCTC-3′ and reverse, 5′-GGGGTTCCCGGTAATCTTTA-3′, 100 bp product). The reaction mixture contained 2.5 mM MgCl_2_, 200 μM dNTP, 1 U Taq DNA polymerase (Invitrogen Corporation, Carlsbad, CA, USA), 100 pmol of specific sense and antisense primers for each gene, and 10 ng of first-strand cDNA in a total volume of 50 μL. The optimal number of cycles within the linear range of amplification was selected. The PCR conditions for amplification were 94°C for 5 min. Thirty-five cycles of 94°C for 45 s, 58.5°C for 45 s, 72°C for 90 s, and 72°C for 7 min using a Mastercycler DNA Engine Thermal Cycler (Eppendorf, Hamburg, Germany). To ensure that equal amounts of reverse-transcribed RNA were added to the PCR, the β-actin housekeeping gene was amplified using oligonucleotides previously reported [37]. The PCR products were analyzed by 10% SDS–polyacrylamide gel electrophoresis and ethidium bromide staining and captured by a Chemidoc XRS + imaging system (BioRad, Hercules, CA, USA). Bands were quantified by scanning densitometry using Image Lab 5.0 software (BioRad, Hercules, CA, USA).

### Western blotting

Ipsilateral dorsal spinal cord segments L1–S1 and DRG (L4–L6) were homogenized in ice-cold lysis buffer (150 mM NaCl, 50 mM Tris, 1 mM EDTA, 1% NP40, 0.1% SDS, 2 mM phenyl-methyl-sulfonyl fluoride, 6.8 µg/mL aprotinin, 4 µg/mL leupeptin, 4 µg/mL pepstatin A, 4 µg/mL soybean trypsin inhibitor and 2 mM NaVO_4_) [35]. Homogenates were centrifuged at 4°C for 10 min at 14,000 rpm, and the supernatant fraction was used to measure protein concentration [38]. Total protein (50 or 100 µg, for spinal cord or DRG, respectively) was resolved by 10% SDS–polyacrylamide gel electrophoresis and transferred to polyvinylidene difluoride membranes. Membranes were blocked with 5% non-fat milk in phosphate-buffered saline at pH 7.4 containing (in mM) (137 NaCl, 2.7 KCl, 10 Na_2_HPO_4_ and 2 KH_2_PO_4_) with Tween 0.05% and they were incubated at 4°C overnight with rabbit anti-bestrophin-1 (ABC-001, 1:200; Alomone Labs, Jerusalem, Israel) or rabbit anti-anoctamin-1 (ACL-011, 1:100; Alomone Labs, Jerusalem, Israel). Horseradish peroxidase-conjugated secondary antibody (anti-rabbit, 711-035-152, 1:6,000; Jackson ImmunoResearch Laboratories Inc, West Grove, PA, USA) was applied for detecting the primary antibody signal using an enhanced chemiluminescence detection system according to the manufacturer´s instructions (Western Lightning Ultra, NEL112001EA, PerkinElmer, Waltham, MA, USA). After bestrophin-1 or anoctamin-1 detection, the membranes were stripped, blocked and incubated with a mouse monoclonal antibody directed against β-actin (MAB1501, 1:10,000; Millipore, Billerica, MA, USA) and its respective horseradish peroxidase-conjugated secondary antibody (anti-mouse, 115-035-003, 1:10,000; Jackson ImmunoResearch Laboratories Inc, West Grove, PA, USA). β-actin expression was used as a loading control to normalize protein expression levels. In addition, antibodies were pre-adsorbed with control bestrophin-1 and anoctamin-1 peptides to validate antibody specificity. Scanning of the immunoblots was performed and the bands were quantified by densitometry using an image analysis program (LabWorks; UVP Inc., Upland, CA, USA).

### Tissue preparation and recording of compound action potential

Rats were anesthetized with a mixture of ketamine (50 mg/kg, i.p.) and xylazine (10 mg/kg, i.p.) 14 days after surgery. A laminectomy was performed while the surgical field was bathed with oxygenated (5% CO_2_ and 95% O_2_) artificial cerebrospinal fluid (CSF) at pH of 7.6 containing (in mM): 117 NaCl, 3.6 KCl, 1.2 MgCl_2_, 2.3 CaCl_2_, 1.2 NaH_2_PO_4_, 25 NaHCO_3_ and 11 glucose at room temperature. The L5 ganglia attached to dorsal root and spinal nerve were removed. The tissue was transferred to a recording chamber and bathed with artificial CSF solution at room temperature.

To evoke action potentials, electrical stimulation (0.3 ms of duration) was applied to the peripheral cut end of the spinal nerve with a suction electrode. The compound action potential (CAP) was recorded in the dorsal root with a suction electrode. The recording electrode was connected to a DC amplifier (World Precision Instruments, Sarasota, FL, USA) with a bandwidth of DC to 10 kHz. Unless otherwise noted, the recordings shown represent the average of 10 stimuli applied every 10 s. The threshold (T) of the faster Aα/β fiber compound action potential (CAP) was determined by increasing the stimulus strength until a visible action potential was evoked 50% of the time. The maximal CAP of the putative C fibers was evoked stimulating at 50xT.

### Drugs

5-Nitro-2-(3-phenylpropylamino)benzoic acid (NPPB), anthracene-9-carboxylic acid (9-AC) and niflumic acid (NFA) were purchased from Sigma-Aldrich (St. Louis, MO, USA). The specific anoctamin-1 inhibitor (T16A_inh_-A01) was purchased from Tocris Bioscience (Avon, Bristol, England). The specific calcium-activated chloride channel inhibitor (CaCC_inh_-A01) was purchased from Merck Millipore (Billerica, MA, USA). All drugs were dissolved in 30% dimethyl sulfoxide (DMSO) in all doses tested.

### Experimental design

In order to determine the role of CaCCs in neuropathic pain, neuropathic (14 days after spinal nerve ligation) and sham animals received an intrathecal (10 μL) or peripheral (into the dorsal surface of the left hind paw, 50 μL) injection of vehicle (30% DMSO) or increasing concentrations of non-selective (NPPB, 9-AC or NFA) or selective (T16A_inh_-A01 or CaCC_inh_-A01) CaCCs inhibitors 5 min before evaluation of tactile allodynia, thermal hyperalgesia or motor coordination. The antiallodynic and antihyperalgesic effects were evaluated for the following 8 h. All behavioral tests were scored by a single investigator who was blind to the treatment received by the subject. Furthermore, in order to assess the role of the CaCCs bestrophin-1 and anoctamin-1 in neuropathic rats, we determined the expression of bestrophin-1 and anoctamin-1 mRNA and protein levels in the ipsilateral dorsal section of the spinal cord and ipsilateral DRG at 1, 7 and 14 days after nerve injury.

As spinal nerve ligation increased anoctamin-1 expression, we next determined its anoctamin-1 mRNA and protein expression in the presence and absence of the most effective CaCCs inhibitors. For this, we intrathecally administered CaCC_inh_-A01 (10 μg), T16A_inh_-A01 (10 μg) or NFA (300 μg) every 6 h for five times starting on day 12 after ligation. Samples of spinal cord and DRG were obtained on day 14 after nerve ligation.

Considering that bestrophin-1 mRNA or protein expression levels were not modified by spinal nerve ligation and the lack of specific inhibitors for this channel, we next determined bestrophin-1 mRNA and protein expression in the presence and absence of its specific antibody. For this study, we intrathecally injected either the anti-bestrophin-1 or anti-anoctamin-1 antibody (2 μg) every 6 h for five times starting on day 12 after ligation, as previously reported for other proteins [39–41]. Withdrawal threshold was evaluated every 6 h after each administration and rats were sacrificed on day 14 after nerve ligation to obtain spinal cord and DRG.

To investigate whether the antiallodynic effect of CaCCs inhibitors was mediated by a reduction of the peripheral nerve injury-induced hyperexcitability, we recorded the C component of the CAP in L5 dorsal roots of naïve, sham and neuropathic rats in the presence and absence of the selective CaCCs inhibitors T16A_inh_-A01 (20 μM) and CaCC_inh_-A01 (20 μM).

### Data analysis and statistics

All behavioral results are reported as the mean ± SEM for six animals/group. Curves were constructed by plotting the paw withdrawal threshold or withdrawal latency as a function of time. An increase of the 50% withdrawal threshold or withdrawal latency was considered as antiallodynic or antihyperalgesic effect, respectively.

In order to determine the dose required to reduce 50% of the possible maximal effect (ED_50_), we calculated the percent of maximum possible effect (%MPE) according to the following equation:

$$\% {\text{MPE}} = \, \left( {{{\left( {{\text{AUC}}_{\text{drug}} - {\text{ AUC}}_{\text{vehicle}} } \right)} \mathord{\left/ {\vphantom {{\left( {{\text{AUC}}_{\text{drug}} - {\text{ AUC}}_{\text{vehicle}} } \right)} {\left( {{\text{AUC}}_{\text{sham}} - {\text{ AUC}}_{\text{vehicle}} } \right)}}} \right. \kern-0pt} {\left( {{\text{AUC}}_{\text{sham}} - {\text{ AUC}}_{\text{vehicle}} } \right)}}} \right) \times \, 100$$

where AUC is the area under the curve of the time-course of the 50% withdrawal threshold per rat of each group. The dose–response curves were constructed by plotting the %MPE versus dose and the experimental points were fitted using least-square linear regression. ED_50_ ± standard error (SE) was calculated according to Tallarida [42].

For mRNA and protein expression, all results are reported as the mean relative intensity ± SEM for three independent animals/group.

For the electrophysiological recordings, all results are reported as the mean of the normalized area under the curve of the C component of the CAP ± SEM for six animals/group.

Statistical differences between two groups were determined by the Student *t* test. One- or two-way analysis of variance (ANOVA), followed by Student–Newman–Keuls or Bonferroni test, were used to compare differences between more than two groups. Differences were considered to reach statistical significance when p < 0.05.

## Results

### CaCCs inhibitors reverse tactile allodynia in spinal nerve ligated rats

**Figure 1:**
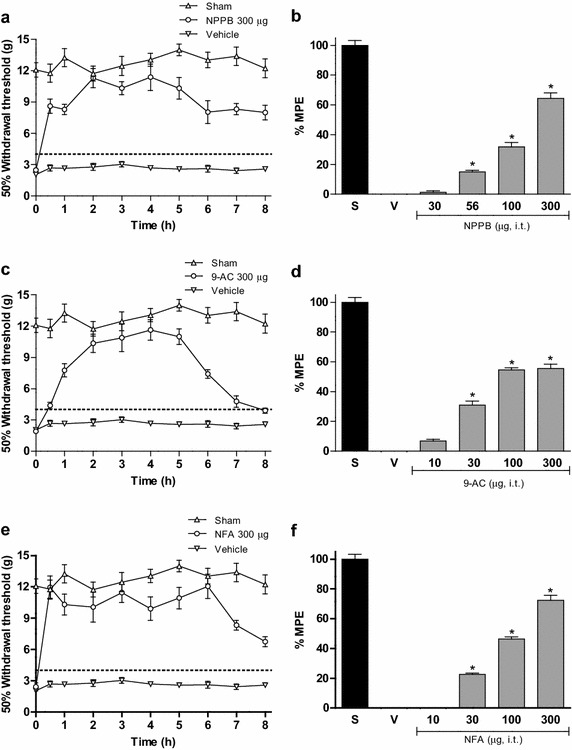
Intrathecal injection of non-selective CaCCs inhibitors reduces tactile allodynia. Time-course of the antiallodynic effect of NPPB (300 μg, **a**), 9-AC (300 μg, **c**) and NFA (300 μg, **e**) in rats subjected to L5/L6 spinal nerve ligation. Withdrawal threshold was assessed 14 days after spinal nerve injury. Dose–response relationship of the antiallodynic effect of NPPB (30–300 μg, **b**), 9-AC (10–300 μg, **d**) and NFA (10–300 μg, **f**) in spinal nerve injured rats compared to sham (S) and vehicle (V) groups. Data are presented as the mean ± SEM for six animals. Note that non-selective CaCCs inhibitors significantly increased withdrawal threshold as well as the % of maximum possible effect (%MPE). *Significantly different from the vehicle group (p < 0.05), as determined by one-way ANOVA followed by the Student–Newman–Keuls test.

**Figure 2:**
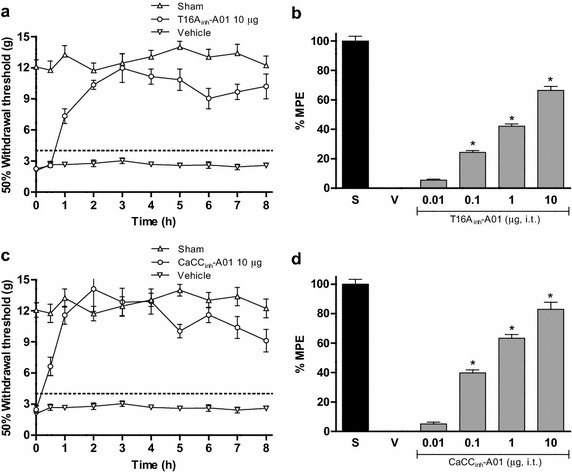
Intrathecal injection of selective CaCCs inhibitors reduces tactile allodynia. Time-course of the antiallodynic effect of T16A_inh_-A01 (10 μg, **a**) and CaCC_inh_-A01 (10 μg, **c**) in rats subjected to L5/L6 spinal nerve injury. Withdrawal threshold was assessed 14 days after spinal nerve injury. Dose–response relationship of the antiallodynic effect of T16A_inh_-A01 (0.01–10 μg, **b**) and CaCC_inh_-A01 (0.01–10 μg, **d**) in spinal nerve injured rats compared to sham (S) and vehicle (V) groups. Data are presented as the mean ± SEM for six animals. Note that selective CaCCs inhibitors significantly increased withdrawal threshold as well as the % of maximum possible effect (%MPE). *Significantly different from the vehicle group (p < 0.05), as determined by one-way ANOVA followed by the Student–Newman–Keuls test.

Ligation of L5/L6 spinal nerves reduced the 50% paw withdrawal threshold response in the ipsilateral paw, as compared to the sham-operated rats, which is indicative of tactile allodynia induction (Figure 1a, c, e; [29]). On the other hand, 14 days after nerve injury intrathecal administration of the non-selective CaCCs inhibitors NPPB, 9-AC or NFA (Figure 1a, c, e), but not vehicle, significantly (p < 0.05) reversed dose-dependently this condition in neuropathic rats (Figure 1b, d, f). Furthermore, spinal, but not peripheral (Additional file 1: Fig. S1), administration of the selective CaCCs inhibitors T16A_inh_-A01 and CaCC_inh_-A01 (Figure 2a, c) had effects similar to those induced by the aforementioned non-specific inhibitors (Figure 2b, d). These declines elicited by the CaCCs inhibitors did not occur in sham-operated rats (Additional file 2: Fig. S2). The maximal antiallodynic effect of these inhibitors in all cases occurred about 2 h after their administration and then decayed gradually in about 8 h. Non-selective CaCCs inhibitors produced a maximal decline of about 65% while the selective CaCCs inhibitors effect reached about 80% of the maximal possible fall. Table 1 lists the ED_50_ of all inhibitors used.

**Table 1:** Effective doses of the non-selective and selective CaCCs inhibitors in spinal nerve injury-induced tactile allodynia. Drugs were administered as a post-treatment. Data were collected from the ipsilateral paw 14 days after spinal nerve ligation. Data are presented as the mean (n = 6) ± SEM.

Inhibitor	ED_50_ (µg)
NPPB	183.2 ± 4.9
9AC	132 ± 46.7
NFA	109.9 ± 3.8
T16A_inh_-A01	1.8 ± 0.3
CaCC_inh_-A01	0.4 ± 0.1

### CaCCs inhibitors reverse thermal hyperalgesia in spinal nerve ligated animals

**Figure 3:**
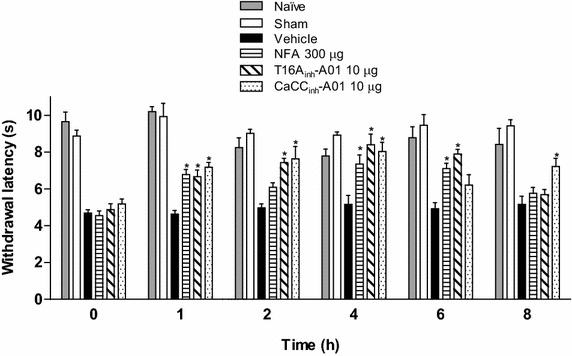
Intrathecal injection of non-selective and selective CaCCs inhibitors reduces thermal hyperalgesia. Time-course of the antihyperalgesic effect of NFA (300 μg), T16A_inh_-A01 (10 μg) and CaCC_inh_-A01 (10 μg) in rats subjected to L5/L6 spinal nerve injury compared to naïve, sham and vehicle groups. Withdrawal latency was assessed 14 days after spinal nerve injury. Data are presented as the mean ± SEM for six animals determined by the mean of three trials for each rat at each time. Note that CaCCs inhibitors increased withdrawal latency. *Significantly different from the vehicle group (p < 0.05), as determined by repeated measures two-way ANOVA followed by the Bonferroni test.

Besides tactile allodynia, ligation of L5/L6 spinal nerves caused a significant decrease in the withdrawal latency time produced by a thermal stress in the ipsilateral, but not contralateral (data not shown), paw of all rats as compared to the naïve or sham-operated animals. On the other hand, intrathecal administration (on day 14th) of either NFA (300 µg), T16A_inh_-A01 (10 µg) or CaCC_inh_-A01 (10 µg), but not vehicle, significantly increased the withdrawal latency time in the ligated animals (Figure 3). The maximal antihyperalgesic effect in all cases occurred in approximately 2 h after drug administration and then decayed gradually in about 8 h.

### Expression of bestrophin-1 and anoctamin-1 in spinal nerve injured rats

**Figure 4:**
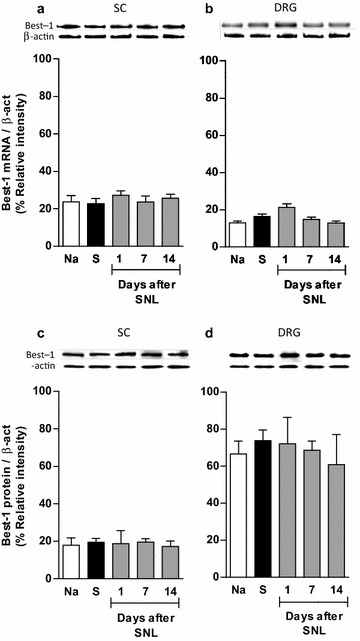
Bestrophin-1 is expressed in the spinal cord and DRG. RT-PCR (**a**, **b**) and western blot (**c**, **d**) analysis of bestrophin-1 (Best-1) at the ipsilateral dorsal portion of the spinal cord (SC) and DRG obtained from naïve (Na), sham (S) and spinal nerve ligated (SNL) rats. Insets show a representative band of the PCR product (*upper panels*) and immunoblot (*lower panels*) obtained with the specific bestrophin-1 and β-actin primers or antibodies, respectively. Data were normalized against β-actin and are expressed as the mean ± SEM of three independent rats. Note that spinal nerve injury did not modify Best-1 mRNA and protein expression.

**Figure 5:**
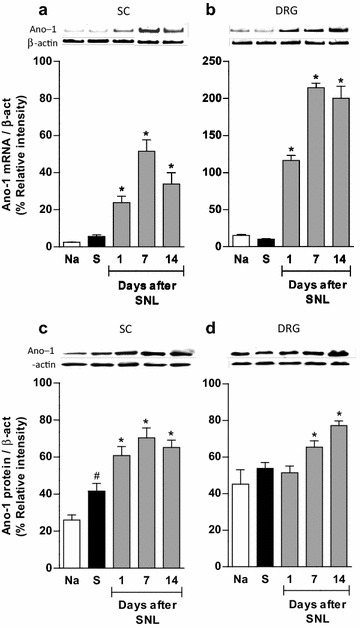
Spinal nerve injury increases anoctamin-1 expression in the spinal cord and DRG. RT-PCR (**a**, **b**) and western blot (**c**, **d**) analysis of anoctamin-1 (Ano-1) at the ipsilateral dorsal portion of the spinal cord (SC) and DRG obtained from naïve (Na), sham (S) and spinal nerve ligated (SNL) rats. Data were normalized against β-actin and are expressed as the mean ± SEM of three independent rats. Insets show a representative band of the PCR product (*upper panels*) and immunoblot (*lower panels*) obtained with the specific anoctamin-1 and β-actin primers or antibodies, respectively. *Significantly different from the S group (P < 0.05), ^#^significantly different from the Na group (P < 0.05), as determined by one-way ANOVA, followed by the Student–Newman–Keuls test. Note that spinal nerve injury enhanced Ano-1 mRNA and protein expression in spinal cord and DRG whereas that surgery increased Ano-1 expression in sham (S) animals in spinal cord but not DRG.

PCR and western blot analysis of the ipsilateral dorsal spinal cord and DRG demonstrated bestrophin-1 (Figure 4) and anoctamin-1 (Figure 5) mRNA and protein expression, in naïve, sham and ligated rats. Western blots resolved bands of about 68 and 90 kDa for bestrophin-1 (Additional file 3: Figure S3A) and anoctamin-1 (Additional file 3: Figure S3B), respectively. On the other hand, immunoreactive bands for both proteins were absent when the primary antibodies were pre-adsorbed with the corresponding antigenic peptides (Additional file 3: Figure S3).

Spinal nerve ligation neither modified bestrophin-1 mRNA and protein expression in the ipsilateral dorsal region of the spinal cord nor in the DRG, as compared to naïve or sham-operated rats (Figure 4). In marked contrast, nerve injury increased (p < 0.05) anoctamin-1 mRNA and protein expression in the dorsal region of the spinal cord and DRG from day 1 to 14 (Figure 5).

### CaCCs inhibitors blunt neuropathy-induced rises on bestrophin-1 and anoctamin-1 mRNA and protein expression

**Figure 6:**
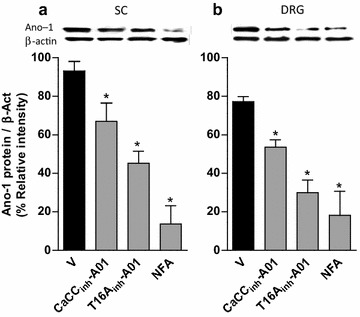
CaCCs inhibition reverses spinal nerve injury-induced rise in anoctamin-1 expression. Western blot analysis of anoctamin-1 (Ano-1) at the ipsilateral dorsal portion of the spinal cord (SC, **a**) and DRG (**b**) obtained from neuropathic rats with repeated intrathecal administration of vehicle (V), CaCC_inh_-A01, T16A_inh_-A01 or NFA. Data were normalized against β-actin and are expressed as the mean ± SEM of three independent rats. *Insets* in **a** and **b** show a representative blot obtained with anoctamin-1 and β-actin primary antibodies. *Significantly different from the V group (p < 0.05), as determined by one-way ANOVA, followed by the Student–Newman–Keuls test. Note that CaCCs inhibitors reduced spinal nerve injury-induced rise in anoctamin-1 expression.

Repeated intrathecal injection (five times, every 6 h starting on day 12 after nerve injury) of CaCC_inh_-A01 (10 μg), T16A_inh_-A01 (10 μg) or NFA (300 μg), but not vehicle, prevented nerve injury-induced rises in anoctamin-1 mRNA (Additional file 4: Figure S4) and protein (Figure 6) expression in the dorsal spinal cord and DRG on day 14 after nerve injury.

**Figure 7:**
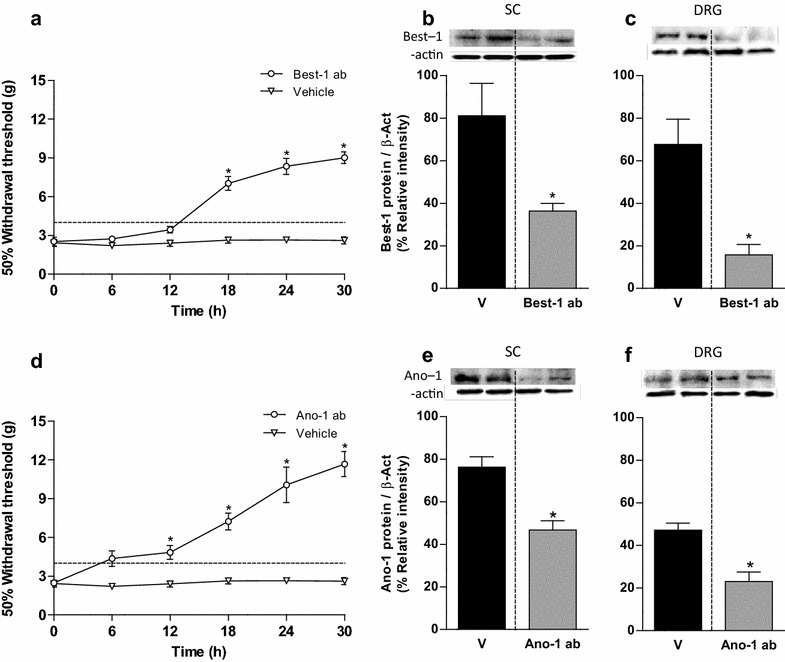
Intrathecal administration of selective antibody anti-bestrophin-1 or anti-anoctamin-1 reverses established allodynia and reduces protein expression. Time-course of the antiallodynic effect of antibodies against bestrophin-1 (Best-1 ab, 2 μg/6 h × 30 h; **a**) or anoctamin-1 (Ano-1 ab, 2 μg/6 h x 30 h; **d**) in rats subjected to L5/L6 spinal nerve ligation. Paw withdrawal threshold was assessed every 6 h starting 12 days after spinal nerve ligation. Data are presented as the mean ± SEM for six animals. *Significantly different from the vehicle group (p < 0.05), as determined by two-way ANOVA followed by the Bonferroni test. Western blot analysis of bestrophin-1 (Best-1, **b**, **c**) or anoctamin-1 (Ano-1, **e**, **f**) at the ipsilateral dorsal portion of the spinal cord (SC) and DRG obtained from neuropathic rats with repeated intrathecal administration of antibodies against bestrophin-1 (Best-1 ab) or anoctamin-1 (Ano-1 ab). Data were normalized against β-actin and are expressed as the mean ± SEM of three independent rats. *Insets* in **b**–**f** show a representative blot obtained with bestrophin-1, anoctamin-1 and β-actin primary antibodies. *Significantly different from the vehicle (V) group (p < 0.05), as determined by the Student *t* test.

Since spinal nerve injury-failure to increase bestrophin-1 expression, we evaluated its involvement in neuropathic pain induction by instead injecting a function blocking specific antibody. Repeated intrathecal anti-bestrophin-1 or anti-anoctamin-1 antibody administration (five times, 2 μg/6 h starting on day 12 after nerve injury), but not vehicle, reversed established tactile allodynia (Figure 7a, d) and this effect was accompanied with a decline of its respective mRNA (Additional file 5: Figure S5) and protein (Figure 7) expression in the dorsal spinal cord and DRG on day 14 after spinal nerve ligation.

### Nerve ligation induced changes in compound action potential (CAP) size and conduction velocity

**Figure 8:**
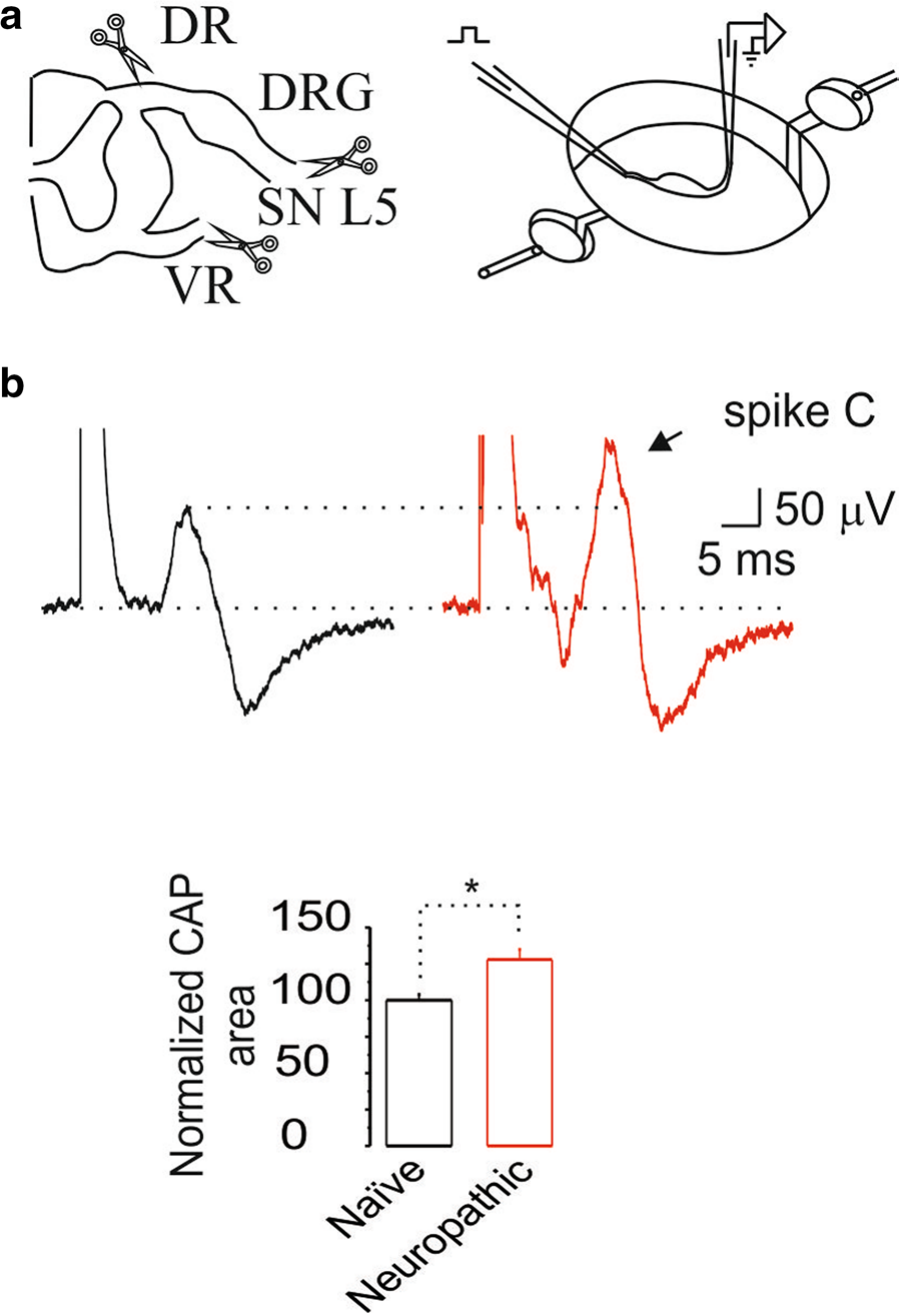
Illustration of the preparation and the compound action potential (CAP). **a** *Left panel* the L5 ganglion attached to dorsal root (DR) and spinal nerve was removed of naïve, sham and neuropathic rats. *Right panel* the tissue was collocated into a recording chamber. The spinal nerve was stimulated at 50xT to evoke maximal C fiber CAP recorded in the dorsal root. **b** *Upper panel* examples of maximal C fiber CAP recorded extracellularly from naïve (*black trace*), sham and neuropathic (*red trace*) rats. The *bar chart* in the *lower panel* shows the comparison of CAP. Note that the size of the CAP in neuropathic was higher than in naïve rats.

In previous studies of mammalian DRG neurons using sharp or patch microelectrodes, action potential duration tended to increase after axotomy [43]. Here, we recorded the C component of the CAP in the dorsal root attached to its ganglion after peripheral stimulation at the spinal nerves (Figure 8a). Area under the curve of the C component of the CAP recorded in neuropathic rats increased in comparison to naïve or sham-operated rats (Figure 8b). Moreover, its conduction velocity was slowed (data not shown).

### Effect of CaCCs blockers on the C component of the CAP

**Figure 9:**
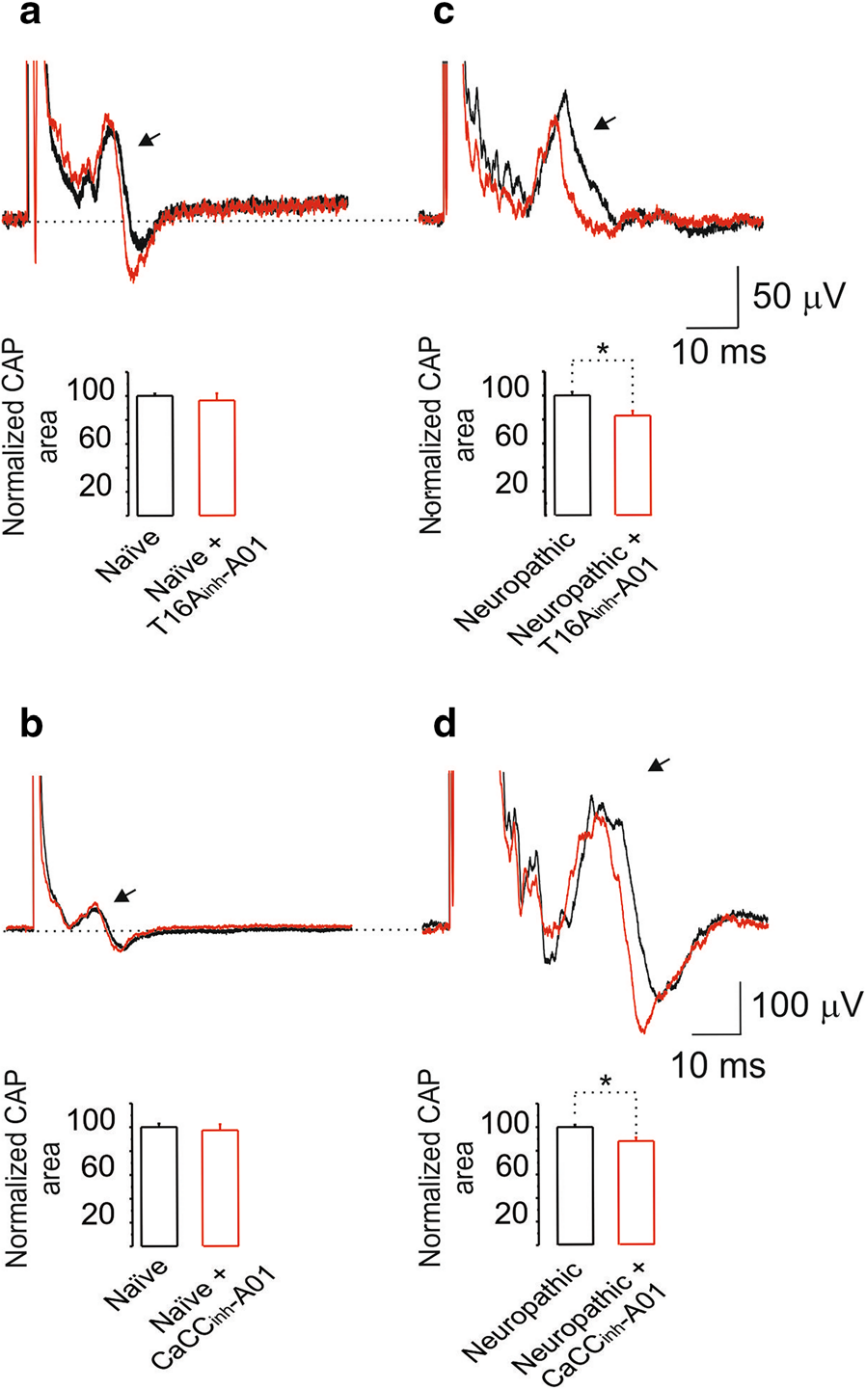
Effect of CaCCs inhibitors on the CAP of naïve and neuropathic rats. *Upper panels* examples of the CAP recorded in absence (control, *black trace*) and in presence of T16A_inh_-A01 [20 μM] (*red trace*) recorded from naïve (**a**) and neuropathic (**c**) rats. The *bar charts* below of traces show the comparison of normalized area under the C component of the CAP between the naïve and T16A_inh_-A01 groups. Note that T16A_inh_-A01 diminished the compound action potential in neuropathic, but not in naïve, rats. *Lower panels* examples of CAP recorded in absence (control, *black trace*) and in presence of CaCC_inh_-A01 [20 μM] (*red traces*) recorded from naïve (**b**) and neuropathic (**d**) rats. The *bar charts* below of traces compare the normalized area under the C component of the CAP between the control and CaCC_inh_-A01 groups. Note that CaCC_inh_-A01 decreased the CAP size in neuropathic, but not in naïve, rats. *Significantly different from the neuropathic group (p < 0.05), as determined by the Student *t* test.

Like other ion channels, CaCCs regulate the excitability of neurons [13]. Application of T16A_inh_-A01 [20 μM] and CaCC_inh_-A01 [20 μM] did not modify the C component of the CAP in the naïve animal preparation (Figure 9a, b). In sharp contrast, these drugs significantly (p < 0.05) reduced the nerve injury-induced rise in the C component of the CAP (Figure 9c, d). These results along with behavioral and molecular studies described above suggest that declines in neuropathic pain induced by CaCCs inhibitors stem from their effects on the action potential of nociceptive primary afferents.

### Assessment of motor coordination

**Figure 10:**
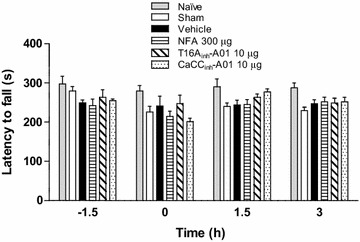
Intrathecal administration of the CaCCs inhibitors did not affect the motor coordination of neuropathic rats. Time-course of the effect of NFA (300 μg), T16A_inh_-A01 (10 μg) or CaCC_inh_-A01 (10 μg) on motor coordination of rats subjected to L5/L6 spinal nerve ligation compared to naïve, sham and vehicle groups. Rats were assessed on the Rotarod apparatus before and at 0, 1.5 and 3 h after drug administration (n = 6). Data are expressed as the latency to fall off the Rotarod apparatus determined by the mean of three trials for each rat at each time. Note that drugs did not modify motor coordination at any time.

Intrathecal administration of either NFA or T16A_inh_-A01 and CaCC_inh_-A01 did not affect motor coordination on the Rotarod apparatus (Figure 10) at the same dosages and times in which they produced the greatest antiallodynic or antihyperalgesic effects.

## Discussion

Our study is the first one to demonstrate that intrathecal administration of both non-selective and selective CaCCs inhibitors reduces tactile allodynia and thermal hyperalgesia in neuropathic rats caused by L5/L6 spinal nerve ligation. First, we found that the non-selective CaCCs inhibitors NPPB, 9-AC and NFA reversed in a dose-dependent manner tactile allodynia. Since these drugs block endogenous CaCCs in vitro [44–47], our data suggest that functional CaCCs participate in the maintenance of neuropathic pain in rats. However, these drugs are non-specific and also block volume-regulated anion channels [48] and K^+^ channels in vitro [49]. To further assess the role of CaCCs in neuropathic pain, we next used the selective anoctamin-1 inhibitor T16A_inh_-A01 [50, 51] as well as the selective CaCCs inhibitor CaCC_inh_-A01 [51, 52]. These drugs also reversed the established tactile allodynia in neuropathic rats. Moreover, NFA, T16A_inh_-A01 or CaCC_inh_-A01 reduced thermal hyperalgesia. These data strongly suggest that CaCC activation contributes to tactile allodynia and thermal hyperalgesia expression. Our data agree with a previous mouse study showing that functional anoctamin-1 ablation reduces spared nerve injury-induced mechanical allodynia and thermal hyperalgesia [21]. Furthermore, our results also indicate that another CaCC, bestrophin, is activated and contributes to neuropathic pain symptomology.

The antiallodynic effectiveness of the CaCCs inhibitors was inversely related to their target selectivity. Namely, the selective anoctamin-1 inhibitor T16A_inh_-A01 was less effective than CaCC_inh_-A01, which inhibits anoctamin-1 and other CaCCs, suggesting that activation of other CaCCs besides anoctamin-1 contribute to inducing neuropathic pain. These results agree with an in vitro study indicating that CaCC_inh_-A01 is a more broad spectrum chloride current inhibitor due to its effectiveness at inhibiting currents mediated by bestrophin-1 and anoctamin-1 activation-1 [51]. On the other hand, T16A_inh_-A01 is less effective since it selectively inhibited chloride currents solely mediated by anoctamin-1 [51, 52]. Other studies demonstrated that local peripheral administration of NFA, NPPB or CaCC_inh_-A01 produced antinociception in rats injected with bradykinin, formalin and carageenan [19, 21, 24]. These data also suggest that CaCCs activation participates in inducing inflammatory pain in rats. Similarly, another indication of CaCC involvement in eliciting neuropathic pain is that bradykinin-induced increases in Ca^2+^-activated chloride currents were prevented by CaCCs inhibitors or siRNA CaCC knockdown [19, 23, 28].

As reported, we found bestrophin-1 and anoctamin-1 mRNA and protein expression in the DRG of naïve rats [19–24]. Furthermore, our results indicate their presence also in the spinal cord. mRNA and protein expression of anoctamin-1 was selectively enhanced by spinal nerve ligation in the dorsal spinal cord and DRG suggesting its involvement in neuropathic pain. This possibility was validated based on our finding that NFA, T16A_inh_-A01 and CaCC_inh_-A01 prevented spinal nerve injury-induced rise in anoctamin-1 mRNA and protein expression and reduced neuropathic pain. Furthermore, repeated administration of the selective antibody against anoctamin-1 (ACL-011, 1:100; Alomone Labs, Jerusalem, Israel) reduced anoctamin-1 expression and tactile allodynia. These data suggest a strong correlation between anoctamin-1 mRNA and protein levels and the presence of allodynia in neuropathic rats. Our results agree with those of García et al. [24], who found that formalin increases anoctamin-1, but not bestrophin-1, protein expression in the rat DRG. It is already known that anoctamin-1 is co-expressed with TRPV1 and IB4 in small sensory neurons [19, 20], and its expression is required for inflammatory and nerve-injury induced thermal hyperalgesia and mechanical allodynia in mice [21]. Taken together, anoctamin-1 plays a pronociceptive role and it is up-regulated by inflammatory [20, 24] and neuropathic (this study) pain in rats.

Unlike us, a previous study found that axotomy solely increases bestrophin-1 transcript expression in mice DRG [23]. This discrepancy may be due to a difference in pain models, species, and molecular methods used in each study. Regarding the first point, it seems that axotomy or spared nerve injury increases chloride currents through enhanced bestrophin-1 expression [23, 28]. In marked contrast, spinal nerve ligation does not modify its expression (our study). Preliminary experiments in our laboratory confirm that spared nerve injury enhances bestrophin-1 expression in rats DRG (data not shown). It seems that axotomy or spared nerve injury increases calcium-activated chloride currents in medium- and large-, but not in small-, diameter neurons displaying regenerative growth [28]. In line with this, bestrophin-1 is preferentially expressed in large and medium size neurons and its increased expression is closely related with nerve regrowth after axotomy [23]. From these data, it appears that axotomy causes more damage and therefore promotes greater nerve regeneration in comparison to spinal nerve ligation. Discrepancies could also be due to the molecular techniques (PCR and western blot versus qPCR and in situ hybridization). Although we could not detect any bestrophin-1 up-regulation, its expression is evident in dorsal spinal cord and DRG of ligated rats. Moreover, we found that administration of a specific anti-bestrophin-1 antibody reduced tactile allodynia and bestrophin-1 expression. This finding reveals that bestrophin-1 participates in the maintenance of neuropathic pain in the spinal nerve ligation model. However, assigning a definitive role for bestrophin-1 in mediating neuropathic pain awaits the future development of selective bestrophin-1 inhibitors.

Besides the antiallodynic effect of CaCCs inhibitors, we found that these drugs partially diminished spinal nerve ligation-induced rise in the C component of the CAP. This finding suggests that nerve injury increases C-like fibers action potential amplitude resulting in more neurotransmitter release at the dorsal horn level. The declines in central sensitization induced by CaCCs inhibitors appear to be associated with their diminution of amplitude enhancement induced by nerve injury. Our data partially agree with previous studies showing that inflammatory mediators increase anoctamin-1-mediated excitability of small sensory neurons [19, 21] while axotomy increases calcium-activated chloride currents in large-, but not small-, diameter neurons [23, 28]. This agreement suggests that spinal nerve ligation increases spinal dorsal roots excitability, which is partially mediated by CaCC activity.

The underlying mechanisms accounting for how nerve injury activates CaCCs are unclear. However, it is known that nerve injury produces peripheral and central sensitization that in turn leads to a massive activation of excitatory mechanisms increasing intracellular Ca^2+^ levels [2]. This response is sufficient to activate CaCCs producing chloride efflux along with inward currents mediating membrane voltage depolarization [53] and hyperexcitability, which then would lead to neuropathic pain.

In summary, the present study revealed that intrathecal administration of non-selective and selective CaCCs inhibitors has antiallodynic and antihyperalgesic effects in spinal nerve injured rats. There is bestrophin-1 and anoctamin-1 mRNA and protein expression in the spinal cord and DRG of neuropathic rats, but only anoctamin-1 is up-regulated after spinal nerve injury. Selective anoctamin-1 blockade reversed increases in its expression induced by spinal nerve injury. Furthermore, intrathecal injection of the selective anti-bestrophin-1 or anti-anoctamin-1 antibody reduced its respective expression along with a reduction of tactile allodynia. Spinal nerve injury increased the C component of the CAP whereas selective CaCCs inhibitors reversed this response. These results strongly suggest that CaCCs, anoctamin-1 and bestrophin-1, participate in the maintenance of neuropathic pain. The identification of CaCC activation as a component of neuropathic pain induction points to the possibility that these channels may be useful targets for treating neuropathic pain in a clinical setting.

## Additional files

Additional file 1:
**Figure S1.** Local peripheral injection of CaCCs inhibitors does not reduce tactile allodynia. Time-course of the effect of local peripheral administration of NFA (300 μg), T16Ainh-A01 (10 μg) and CaCCinh-A01 (10 μg) in rats subjected to L5/L6 spinal nerve ligation. Withdrawal threshold was assessed 14 days after spinal nerve injury. Data are presented as the mean ± SEM for 6 animals. *Significantly different from the vehicle group (p<0.05), as determined by repeated measures two-way ANOVA followed by the Bonferroni test. Additional file 2:
**Figure S2.** Intrathecal injection of CaCCs inhibitors in sham-operated rats does not affect withdrawal threshold. Time-course of the effect of intrathecal injection of NFA (300 μg), T16Ainh-A01 (10 μg) and CaCCinh-A01 (10 μg) in sham-operated rats. Withdrawal threshold was assessed 14 days after sham surgery. Data are presented as the mean ± SEM for 6 animals. Note that CaCCs inhibitors did not have any effect on withdrawal threshold. Additional file 3:
**Figure S3.** Pre-adsorption of bestrophin-1 and anoctamin-1 with the corresponding control peptide. Western blot analysis of bestrophin-1 (Best-1, panel A) and anoctamin-1 (Ano-1, panel B) at the ipsilateral DRG obtained from neuropathic rats incubated with the selective antibody (control) and pre-adsorbed with the corresponding control peptide (PEP). Data were normalized against β-actin and are expressed as the mean ± SEM. of 3 independent rats. * Significantly different from the control group (p<0.05), as determined by the Student *t* test. Insets in A and B show representative blots obtained with bestrophin-1, anoctamin-1 and β-actin primary antibodies which revealed bands around 68-, 90- and 43-kDa, respectively. Additional file 4:
**Figure S4.** CaCCs inhibition reduces spinal nerve injury-induced rise in anoctamin-1 mRNA expression. RT-PCR analysis of anoctamin-1 (Ano-1) at the ipsilateral dorsal portion of the spinal cord (SC, panel A) and DRG (panel B) obtained from neuropathic rats with repeated intrathecal administration of vehicle (V), CaCC_inh_-A01, T16A_inh_-A01 or NFA. Data were normalized against β-actin and are expressed as the mean ± SEM of 3 independent rats. *Significantly different from the vehicle group (p<0.05), as determined by one-way ANOVA, followed by the Student-Newman-Keuls test. Insets in A and B show representative bands obtained with anoctamin-1 and β-actin primers. Additional file 5:
**Figure S5.** CaCCs inhibition by antibodies reduces spinal nerve injury-induced rise in bestrophin-1 and anoctamin-1 mRNA expression. RT-PCR analysis of bestrophin-1 (Best-1, panels A and B) or anoctamin-1 (Ano-1, panels C and D) at the ipsilateral dorsal portion of the spinal cord (SC) and DRG obtained from neuropathic rats with repeated intrathecal administration of antibodies against bestrophin-1 (Best-1 ab) or anoctamin-1 (Ano-1 ab). Data were normalized against β-actin and are expressed as the mean ± SEM. of 3 independent rats. *Significantly different from the vehicle group (p<0.05), as determined by the Student *t* test. Insets show representative bands obtained with bestrophin-1, anoctamin-1 and β-actin primers.

## Abbreviations

9-AC: anthracene-9-carboxylic acid
ANOVA: analysis of variance
CaCCs: calcium-activated chloride channels
CAP: compound action potential
CSF: cerebrospinal fluid
DRG: dorsal root ganglia
DRR: dorsal root reflexes
ED_50_: effective dose 50
kDa: kilodaltons
MPE: maximum possible effect
mRNA: messenger ribonucleic acid
NFA: niflumic acid
NPPB: 5-nitro-2-(3-phenylpropylamino)benzoic acid
PAD: primary afferent depolarization
PBS: phosphate-buffered saline
PCR: polymerase chain reaction
qPCR: quantitative polymerase chain reaction
SEM: standard error of the mean
