# Supplementary material for: Role of anoctamin-1 and bestrophin-1 in spinal nerve ligation-induced neuropathic pain in rats
Source: Mol Pain. 2015 Jul 1;11:41. doi: 10.1186/s12990-015-0042-1 (PMC4487556; doi:10.1186/s12990-015-0042-1)
Supplement: Additional file 1: — Figure S1. Local peripheral injection of CaCCs inhibitors does not reduce tactile allodynia. Time-course of the effect of local peripheral administration of NFA (300 μg), T16Ainh-A01 (10 μg) and CaCCinh-A01 (10 μg) in rats subjected to L5/L6 spinal nerve ligation. Withdrawal threshold was assessed 14 days after spinal nerve injury. Data are presented as the mean ± SEM for 6 animals. *Significantly different from the vehicle group (p<0.05), as determined by repeated measures two-way ANOVA followed by the Bonferroni test. [file 12990_2015_42_MOESM1_ESM.docx]

Fig. S1. Pineda-Farias et al.
