# Supplementary material for: Role of anoctamin-1 and bestrophin-1 in spinal nerve ligation-induced neuropathic pain in rats
Source: Mol Pain. 2015 Jul 1;11:41. doi: 10.1186/s12990-015-0042-1 (PMC4487556; doi:10.1186/s12990-015-0042-1)
Supplement: Additional file 2: — Figure S2. Intrathecal injection of CaCCs inhibitors in sham-operated rats does not affect withdrawal threshold. Time-course of the effect of intrathecal injection of NFA (300 μg), T16Ainh-A01 (10 μg) and CaCCinh-A01 (10 μg) in sham-operated rats. Withdrawal threshold was assessed 14 days after sham surgery. Data are presented as the mean ± SEM for 6 animals. Note that CaCCs inhibitors did not have any effect on withdrawal threshold. [file 12990_2015_42_MOESM2_ESM.docx]

Fig. S2. Pineda-Farias et al.
