# Supplementary figures and images for: Role of anoctamin-1 and bestrophin-1 in spinal nerve ligation-induced neuropathic pain in rats
Source: Mol Pain. 2015 Jul 1;11:41. doi: 10.1186/s12990-015-0042-1 (PMC4487556; doi:10.1186/s12990-015-0042-1)

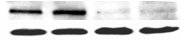

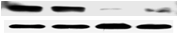


**** 90 kDa

** **43 kDa

**** 68 kDa

** **43 kDa

Ano–1

β-actin

Best–1

β-actin

Fig. S3. Pineda-Farias et al.

Supplement: Additional file 3: — Figure S3. Pre-adsorption of bestrophin-1 and anoctamin-1 with the corresponding control peptide. Western blot analysis of bestrophin-1 (Best-1, panel A) and anoctamin-1 (Ano-1, panel B) at the ipsilateral DRG obtained from neuropathic rats incubated with the selective antibody (control) and pre-adsorbed with the corresponding control peptide (PEP). Data were normalized against β-actin and are expressed as the mean ± SEM. of 3 independent rats. * Significantly different from the control group (p<0.05), as determined by the Student t test. Insets in A and B show representative blots obtained with bestrophin-1, anoctamin-1 and β-actin primary antibodies which revealed bands around 68-, 90- and 43-kDa, respectively. [file 12990_2015_42_MOESM3_ESM.docx]

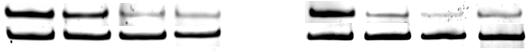


Ano–1

β-actin

SC

DRG

Fig. S4. Pineda-Farias et al.

Supplement: Additional file 4: — Figure S4. CaCCs inhibition reduces spinal nerve injury-induced rise in anoctamin-1 mRNA expression. RT-PCR analysis of anoctamin-1 (Ano-1) at the ipsilateral dorsal portion of the spinal cord (SC, panel A) and DRG (panel B) obtained from neuropathic rats with repeated intrathecal administration of vehicle (V), CaCCinh-A01, T16Ainh-A01 or NFA. Data were normalized against β-actin and are expressed as the mean ± SEM of 3 independent rats. *Significantly different from the vehicle group (p<0.05), as determined by one-way ANOVA, followed by the Student-Newman-Keuls test. Insets in A and B show representative bands obtained with anoctamin-1 and β-actin primers. [file 12990_2015_42_MOESM4_ESM.docx]

DRG

SC


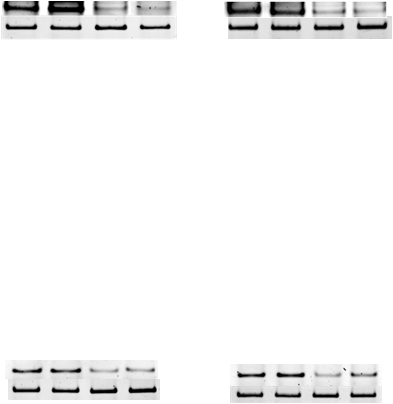


Best–1

β-actin

DRG

SC

Ano–1

β-actin

Fig. S5. Pineda-Farias et al.

Supplement: Additional file 5: — Figure S5. CaCCs inhibition by antibodies reduces spinal nerve injury-induced rise in bestrophin-1 and anoctamin-1 mRNA expression. RT-PCR analysis of bestrophin-1 (Best-1, panels A and B) or anoctamin-1 (Ano-1, panels C and D) at the ipsilateral dorsal portion of the spinal cord (SC) and DRG obtained from neuropathic rats with repeated intrathecal administration of antibodies against bestrophin-1 (Best-1 ab) or anoctamin-1 (Ano-1 ab). Data were normalized against β-actin and are expressed as the mean ± SEM. of 3 independent rats. *Significantly different from the vehicle group (p<0.05), as determined by the Student t test. Insets show representative bands obtained with bestrophin-1, anoctamin-1 and β-actin primers. [file 12990_2015_42_MOESM5_ESM.docx]
